# Treatment‐Line‐Specific Outcomes of Obinutuzumab in Membranous Nephropathy: A Systematic Review and Meta‐Analysis

**DOI:** 10.1155/ijne/6372570

**Published:** 2026-11-09

**Authors:** Abdelrahman M. Elettreby, Ahmed A. Abo Elnaga, Faisal A. Al-Harbi, Sayed Sayem, Tamara Aburiash, Alam Eldin Musa Mustafa, Dina Khodeer, Ahmed Mohamed Aboelkheir, Mostafa Gamal

**Affiliations:** ^1^ Faculty of Medicine Mansoura University Mansoura Egypt mans.edu.eg; ^2^ Department of Medicine King Faisal Specialist Hospital and Research Centre Riyadh Saudi Arabia kfshrc.edu.sa; ^3^ Department of Statistics Comilla University Cumilla 3506 Bangladesh cou.ac.bd; ^4^ Faculty of Medicine An-Najah National University Nablus State of Palestine najah.edu; ^5^ Department of Child Health College of Medicine King Khalid University Abha Saudi Arabia kku.edu.sa; ^6^ Department of Pharmacology College of Medicine Imam Mohammad Ibn Saud Islamic University (IMSIU) Riyadh 13317 Saudi Arabia imamu.edu.sa; ^7^ Clinical Nephrology and Kidney Transplantation Department Urology and Nephrology Center Mansoura University Mansoura Egypt mans.edu.eg

**Keywords:** anti-CD20, membranous nephropathy, obinutuzumab, PLA2R antibodies, rituximab refractory

## Abstract

**Background:**

Primary membranous nephropathy (PMN) is an autoimmune glomerulonephritis triggered by B‐cell dysfunction, where rituximab achieved clinical remission in about 55%–65% of patients, while 35%–45% of patients are nonresponders. Obinutuzumab, a humanized type II anti‐CD20 monoclonal antibody that showed more profound B‐cell depletion, may improve treatment outcomes in both treatment‐naïve and refractory patients. This systematic review and meta‐analysis evaluate the clinical and immunological outcomes of obinutuzumab in PMN.

**Methods:**

We performed a systematic literature search across PubMed, Scopus, and Web of Science through January 2026 to identify studies assessing the effect of obinutuzumab in PMN. We performed meta‐analyses using the meta package in R software.

**Results:**

As first‐line therapy, pooled complete response was 0.324, rising from 0.257 at 6 months to 0.485 at 12 months, and partial response was 0.508. Clinical remission and immunological response were both high ranging between 81% and 89%. As second‐line therapy after rituximab, complete response was lower (18.1%) but partial response remained high (54.7%), with clinical remission increasing to 83.5% at 12 months and immunological response improving significantly to 90.5% at 12 months. In rituximab‐refractory patients, complete and partial response rates were 27.8% and 54.3%, respectively, with clinical remission improving notably by 12 months (84.5%). Secondary analyses showed increased serum albumin and reduced proteinuria over time.

**Conclusion:**

Obinutuzumab was associated with clinical and immunological remission in PMN. Clinical and immunological responses were observed across treatment‐naïve, previously treated, and rituximab‐refractory populations, though differences between these nonrandomized treatment‐line groups should not be interpreted as comparative efficacy. No clear short‐term change in kidney‐function measures was detected.

## 1. Introduction

Primary membranous nephropathy (PMN) is a significant etiology for adult nephrotic syndrome. It contributes substantially to morbidity and increases the risk of long‐term kidney damage and disease progression. PMN is a disease of podocytes since autoantibodies, primarily IgG4, target podocyte antigens, leading to the formation of subepithelial immune complexes that trigger complement‐mediated injury and disruption of the glomerular filtration mechanism [1, 2]. Identification of the podocyte antigen, the M‐type phospholipase A2 receptor (PLA2R), revolutionized the diagnostic and monitoring landscape. PLA2R antibodies can be detected in a significant portion of PMN and correlate with the glomerular subepithelial immune deposits [3, 4].

Patients with PMN experience clinical remission; however, others develop a range of complications associated with persistent nephrotic syndrome and progressive kidney dysfunction. In PMN, nephrotic syndrome by itself poses a risk of infections, dyslipidemia, thromboembolism, the risk of which is higher in PMN than in other nephrotic causes, which can be explained by the levels of hypoalbuminemia present [5, 6]. Anti‐PLA2R antibodies titers are indicative of immune activity, and changing levels of these antibodies can signal the potential for spontaneous remission and inform strategies for therapeutic escalation [7, 8]. Clinical evidence of remission, particularly in the proteinuria levels, can be delayed from the reduction in the immunological response, which is associated with the time needed for repair of the podocytes and basement membrane after the autoimmune process is controlled [9].

The current guidelines recommend initiating optimized supportive care as the first step and only incorporates immunosuppression for patients with persistent nephrotic syndrome or at higher risk for progression. The KDIGO guidelines recommend individualized treatments according to therapeutic objectives and comorbidities, including rituximab, cyclophosphamide plus glucocorticoids, or calcineurin inhibitor (CNI)–based regimens [10]. CNIs are also good options for rapid reduction of proteinuria, but carry the risks of nephrotoxicity and frequent relapses after withdrawal [11, 12]. The use of rituximab in comparison to cyclosporine has documented benefits for long‐lasting remission and has guided contemporary clinical practice [13]. Nonetheless, a clinically significant number of patients had little or no response, some with significant delays, and others with relapses, especially those with high immunologic activity, prior immunosuppression exposure, or prior anti‐CD20 treatment. Recent studies reported that the use of a single immunosuppressive regimen will not promote sustained remission in the majority of cases, underscoring the need for therapies that are less toxic and can promote extensive immunologic control, especially in refractory case scenarios [14, 15].

Obinutuzumab is a type II humanized glycoengineered monoclonal antibody that targets CD20 B lymphocytes, allowing for B‐lymphocyte depletion and suppression of pathogenic autoantibodies. Obinutuzumab is designed to optimize antibody‐dependent cellular phagocytosis and to promote direct apoptosis in B cells, compared with type I anti‐CD20 agents such as rituximab [16, 17]. The current literature has rapidly expanded from early case reports to real‐world cohorts and comparative studies on PMN patients [18, 19]. However, the evidence is still scattered across varying lengths of follow‐up, and differing definitions of clinical remission with uncertainty about expected remission. Therefore, we conducted this systematic review and meta‐analysis to investigate the role of obinutuzumab in patients with PMN.

## 2. Methods

### 2.1. Protocol and Registration

The protocol was prospectively registered in PROSPERO (Registration ID: CRD420261321565). This study followed the Preferred Reporting Items for Systematic Reviews and Meta‐Analyses (PRISMA) statement guidelines [20].

### 2.2. Information Sources and Search Strategy

We performed a literature search across PubMed, Scopus, and Web of Science from their inception up to January 2026 to identify relevant studies using the following search strategy: (“Obinutuzumab” OR obinutuzumab OR GA101 OR Gazyva^∗^ OR Gazyvaro OR “anti‐CD20”) AND (“Glomerulonephritis, Membranous” OR “membranous nephropathy^∗^” OR “membranous glomerulonephritis” OR PLA2R OR “phospholipase A2 receptor” OR THSD7A). To be pragmatic, we did not apply any filters or restrictions to avoid limiting our search.

### 2.3. Eligibility Criteria

#### 2.3.1. Inclusion Criteria

Eligibility criteria were prespecified, including studies fulfilling the following PICO framework—(i) *Population*: Adult patients with membranous nephropathy (either treatment naïve, with prior immune suppressive or rituximab resistant) diagnosed by kidney biopsy or a compatible clinicopathologic phenotype supported by serology. (ii) *Intervention*: Obinutuzumab of different doses, and schedules, administered as first‐line immunosuppressive therapy or as treatment for previously treated, relapsed, or rituximab‐refractory PMN. First‐line was defined as no previous immunosuppressive therapy before obinutuzumab; second‐line of therapy means previous nonspecific immunosuppressive therapy or relapse/insufficient response; rituximab‐refractory refers to previous rituximab exposure with failure to achieve CR/PR, persistent nephrotic‐range proteinuria, and/or persistent anti‐PLA2R positivity according to each study definition. (iii) *Comparator*: Studies were eligible with or without a comparator group. (iv) *Outcomes*: At least one prespecified outcome: clinical response/remission (complete remission [CR], partial remission [PR], or composite clinical remission) at any follow‐up time point; immunological response and changes in proteinuria and serum albumin; kidney function measures (estimated glomerular filtration rate [eGFR]/serum creatinine).

#### 2.3.2. Exclusion Criteria

We excluded studies that (1) did not report on obinutuzumab‐treated patients with membranous nephropathy, (2) pertained only to secondary MN without any primary MN data, (3) did not report on clinical or immunologic outcomes, and (4) nonpeer‐reviewed records (e.g., conference abstracts without complete data), reviews, editorials, or preclinical studies, and case reports less than five cases, studies with duplicate or overlapping cohorts in which no other data could be utilized.

### 2.4. Study Selection

All retrieved records from the database search were imported into EndNote [21] for deduplication before screening. The updated deduplicated library was exported to the Rayyan platform for blinded screening [22]. Two authors (AAA and AME) screened the titles and abstracts independently against the prespecified criteria. Citations considered potentially relevant were moved to the full‐text assessment. The same reviewers evaluated the full text independently to verify eligibility and documented the reasons for exclusion at the full‐text level. Any discrepancies at any stage were discussed to reach a consensus and, if necessary, a third reviewer was called in.

### 2.5. Data Extraction and Outcome Measures

Two independent authors (AME and AMA) extracted pertinent data from each included study into a uniform, prepiloted online sheet after a calibration meeting between them. Items extracted included summary items of included studies (study name, year of publication, sample size, location, population details, intervention and control details, concurrent treatments, previous therapies, as well as follow‐up period), baseline parameters of enrolled patients (age, sex, time from disease to obinutuzumab onset, concomitant diseases, eGFR, serum creatinine, 24 h proteinuria, serum albumin, and anti‐PLA2R antibody levels).

### 2.6. Outcome Definitions

The primary outcomes were CR, PR, clinical remission rate (CRR), and immunological response after obinutuzumab therapy. CR was defined as proteinuria < 0.3 g/24 h (or equivalent UPCR/uPCR < 300 mg/g) plus stable kidney function. PR was defined as a ≥ 50% reduction in proteinuria from baseline, with achievement of subnephrotic proteinuria < 3.5 g/24 h (UPCR < 3500 mg/g) plus stable kidney function. CRR was defined as the proportion of patients achieving clinical remission (CR or PR) at the prespecified follow‐up time point(s): CRR = (number with CR + number with PR)/total evaluable patients. IR = anti‐PLA2R negativity according to each study cut‐off. Stable kidney function was defined as no significant clinical deterioration, such as no serum creatinine doubling and no major eGFR decline. Secondary outcomes included change from baseline in serum albumin (g/L), protein in urine (g/24 h), and kidney function indices (including serum creatinine [μmol/L] and eGFR [ml/min/1.73 m^2^]).

Due to the clinical heterogeneity in treatment context, we executed individual, stratified meta‐analyses by treatment line: (1) obinutuzumab as first‐line immunosuppressive therapy, (2) obinutuzumab as second‐line therapy in patients with prior exposure to immunosuppressive treatment, and (3) obinutuzumab in rituximab‐refractory membranous nephropathy. This classification was operational and did not imply a clinically homogeneous population; patients classified as first‐line varied in disease duration, duration of supportive care, baseline proteinuria, and anti‐PLA2R activity, risk category, and the clinical threshold for initiating immunosuppression.

### 2.7. Risk of Bias Assessment

Two reviewers independently assessed the risk of bias (methodological quality) using the Newcastle Ottawa tool for Cohort Studies [23] for cohort designs and the JBI Critical Appraisal Checklist for Case Series [24] for case series.

### 2.8. Statistical Analysis and Heterogeneity

We performed meta‐analyses of single‐arm outcomes to estimate pooled outcome proportions and within‐study changes from baseline of obinutuzumab in membranous nephropathy, stratified a priori into three clinical subgroups: (1) first‐line obinutuzumab, (2) second‐line obinutuzumab after prior immunosuppressive therapy, and (3) rituximab‐refractory disease. The treatment‐line strata were synthesized separately for descriptive purposes. No formal comparison of treatment efficacy across treatment‐line strata was performed, and differences between strata should not be interpreted as evidence that treatment line modifies the effect of obinutuzumab. For rituximab‐refractory PMN, the primary pooled estimates were restricted to retrospective Chinese studies to avoid combining geographically and methodologically distinct retrospective data with the single Italian prospective cohort. For dichotomous endpoints (complete response, partial response, clinical remission, and immunological response), study‐specific proportions were pooled using the meta package in R [25], applying the arcsine square root transformation and an inverse‐variance weighting approach. Random‐effects models were used, with between‐study variance estimated using restricted maximum likelihood. To improve the reliability of confidence intervals in the presence of few studies and heterogeneity, we used the Hartung–Knapp adjustment. Where applicable, analyses were stratified by follow‐up time point using subgrouping. For continuous outcomes (serum albumin, proteinuria, serum creatinine, and eGFR), we synthesized within‐study changes from baseline using inverse‐variance random‐effects models and reported pooled mean differences (MD) with 95% CI. Because the available evidence was predominantly from single‐arm designs, the meta‐analysis did not estimate adjusted comparative treatment effects and did not adjust for baseline differences between treatment groups. For continuous outcomes, we pooled within‐study mean changes from baseline as mean differences (MDs) in their original clinical units after unit harmonization, rather than standardized mean differences, to preserve clinical interpretability. To reduce incomparability caused by different measurement schedules, analyses were stratified by follow‐up time point whenever data allowed. Between‐study heterogeneity was quantified using the *I* ^2^ statistic and *τ* ^2^, with Cochran’s *Q* test *p* values, and subgroup differences were evaluated using a chi‐square test for subgroup differences. Small‐study effects publication bias were assessed using Doi plots with the Luis Furuya–Kanamori (LFK) index.

## 3. Results

### 3.1. Study Selection

The database search identified 351 relevant records. After removing 137 duplicates, 214 records were screened, of which 188 were excluded. Twenty‐six full‐text reports were sought and successfully retrieved. After full‐text assessment of these 26 records based on eligibility criteria, 15 studies [18, 19, 26–38] were eligible for this review after exclusion of 11 articles (Figure 1).

**FIGURE 1:**
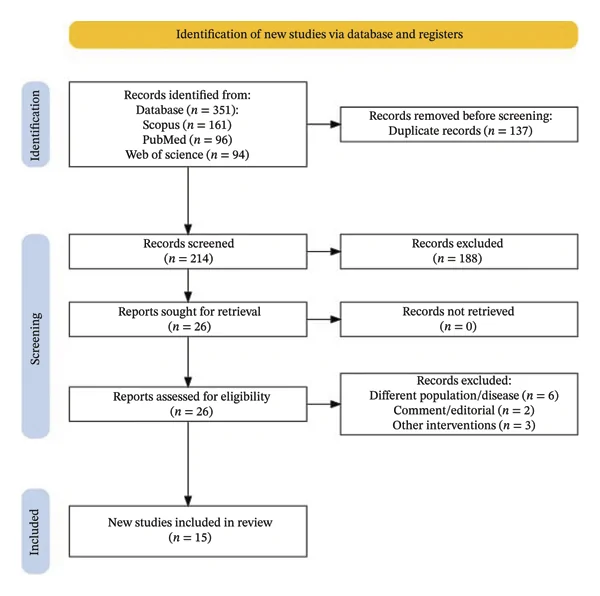
PRISMA flow diagram of the studies’ selection process.

### 3.2. Characteristics of Included Studies

Across 15 included studies conducted mainly in China, with one prospective cohort from Italy, participants largely reflected clinically challenging PMN, either refractory or nonresponsive disease, rituximab‐resistant patients, or some cohorts evaluated obinutuzumab as first‐line therapy. Sample sizes were generally small across studies, with follow‐up ranging between 6 and 24 months (Table 1). Baseline characteristics were broadly consistent with nephrotic syndrome: mean age clustered in middle‐to‐late adulthood, a male predominance in most cohorts, and high PLA2R positivity in many studies. Kidney function at baseline was generally preserved across cohorts (eGFR commonly around 80–100 mL/min/1.73 m^2^), while proteinuria and hypoalbuminemia reflected substantial disease activity. Prior or concomitant immunosuppression was frequent, particularly rituximab exposure, CNIs, cyclophosphamide, and glucocorticoids (Table 2).

**TABLE 1:** Summary of the included studies.

Study ID	Study design	Location	Population	Intervention (I)	Comparator (C)	Concurrent treatment	Previous therapies	Follow‐up, months	Sample size	
									I	C
Xue Li (2025)	Retrospective study	China	Adult patients aged between 18 and 80 years old diagnosed with PLA2R‐associated primary membranous nephropathy with eGFR of at least 30 mL/min/1.73 m^2^	Obinutuzumab 1000 mg in 1000 mL saline infused over 11 h (50 mg/h for 2 h then 100 mg/h for 9 h), repeated after 2–3 weeks, with dexamethasone + promethazine premedication to prevent infusion reactions	Rituximab	NA	NA	6	25	22

Su (2025)	Retrospective study	China	Adult patients aged over 18 years old diagnosed with PLA2R‐associated membranous nephropathy	Obinutuzumab 1 g of two doses (2 weeks apart), readjusted at 6 months to 1–2 g if proteinuria > 3.5 g/day, with steroid/promethazine/indomethacin premedication before infusion in 500 mL normal saline to minimize reactions	NA	Rivaroxaban and lipid‐lowering therapy	Rituximab, cyclophosphamide, and MMF	9.25 ± 4.2	20	NA

Huixian Li (2025)	Case series	China	Adult patients with PLA2R‐associated membranous nephropathy	Obinutuzumab (at least two 1 g doses) 2 weeks apart with dexamethasone 20 mg and diphenhydramine 25 mg for reactions combined with rituximab IV as 1 g for two doses 2 weeks apart	NA	Rituximab	Immunosuppressants (glucocorticoid, calcineurin inhibitors, rituximab, cyclophosphamide, and MMF)	12.6 ± 5.0	17	NA

Xu (2025)	Retrospective study	China	Adult patients with refractory membranous nephropathy with symptoms of nephrotic syndrome	Obinutuzumab 1 g IV (1‐2 doses, ≥ 2‐week interval) with methylprednisolone + ebastine + acetaminophen premedication for infusion reaction	Rituximab IV either 375 mg/m^2^ (1–4 doses, ≥ 1‐week interval) or 1 g (1–2 doses, ≥ 2‐week interval) with dexamethasone + diphenhydramine premedication	ACEI, ARB, diuretics, lipid‐lowering drugs, and prophylactic antithrombotic treatment	Glucocorticoids, calcineurin inhibitors, and cyclophosphamide	14.23 ± 10.9	20	31

Luyao Li (2025)	Retrospective study	China	Adult patients with refractory idiopathic membranous nephropathy	Obinutuzumab was given as 1000 mg per dose, with the cycle and dosing interval individualized by the physician according to patient’s condition	NA	52% of patients received immunosuppressive drugs (e.g., steroid, cyclophosphamide) during study	Rituximab, prednisone, cyclophosphamide, cyclosporine, tacrolimus, MMF	12.67 ± 1.57	25	NA

Shi (2025)	Retrospective study	China	Adult patients with PLA2R‐associated membranous nephropathy	Standard two‐dose regimen of obinutuzumab (1 g given at intervals of 2 weeks) + 1 g oral paracetamol, 25 mg oral chlor‐trimeton and 20 mg dexamethasone IV as premedication G1: obinutuzumab as initial therapy, G2: obinutuzumab for resistant to therapy	NA	ARBs and statins	Rituximab and immunosuppressives	12	G1: 27, G2: 23	

Cao (2025)	Retrospective study	China	Adult histologically negative patients for PLA2R with primary membranous nephropathy	Obinutuzumab 1 g IV every 2 weeks for 2 doses. Premedication 30 min before infusion included IV methylprednisolone 80 mg, oral ebastine 10 mg, and oral acetaminophen 600 mg	Rituximab either 375 mg/m^2^ IV weekly for 4 doses or 1 g IV every 2 weeks for 2 doses with similar premedications	Supportive therapy, including ACEIs, ARBs, diuretics, and lipid‐lowering medications	NA	12	18	21

Su (2024)	Retrospective study	China	Adult patients with primary membranous nephropathy	Obinutuzumab infusions of 1000 mg diluted in 500 mL normal saline as initial line or second line with 1–4 doses according to clinician consultation infused at rate of 10 mL/h for 30 min, then increased every 30 min up to a maximum of 200 mL/h with ibuprofen, IV dexamethasone and IM diphenhydramine for reactions	NA	NA	Glucocorticoid, calcineurin inhibitors, rituximab, cyclophosphamide, triptolide, and MMF	9.5 ± 2.65	59	NA

Xu (2024)	Retrospective study	China	Adult patients with primary membranous nephropathy during COVID‐19	Obinutuzumab 1 g IV (1–2 doses, ≥ 2 weeks apart) with premedication 30 min before using IV methylprednisolone 80 mg + oral ebastine 10 mg + oral acetaminophen 600 mg	Rituximab	Immunosuppressive drugs (glucocorticoids and calcineurin inhibitors)	Glucocorticoids, cyclophosphamide, rituximab, and calcineurin inhibitors	NA	18	46

Lin (2024)	Retrospective study	China	Adult patients with primary refractory membranous nephropathy	Obinutuzumab 1 g, with additional 1 g was repeated every 2 weeks if B‐cell depletion was not achieved	NA	NA	Calcineurin inhibitors, rituximab, cyclophosphamide, prednisone, ARB, and MMF	13.6	18	NA

Chen (2025)	Retrospective study	China	Adult patients with refractory membranous nephropathy	Obinutuzumab 1 g IV (≥ 6 months after rituximab), diluted in 500 mL saline (25 mL/h for 30 min then escalated every 30 min up to 200 mL/h based on tolerance infusion rate), with steroid + promethazine premedication	NA	NA	Prednisone, cyclophosphamide, cyclosporine, tacrolimus and rituximab	10.17 ± 5.05	7	NA

Hao (2024)	Case series	China	Adult patients with primary membranous nephropathy	Two IV doses of obinutuzumab 1000 mg given 2 weeks apart, with premedication (dexamethasone IV 1 h before, ibuprofen PO + diphenhydramine IM 30 min before) to prevent infusion reactions as initial therapy	NA	Supportive care (losartan 50–100 mg)	NA	6	12	NA

Liu (2025)	Case series	China	Adult patients with primary membranous nephropathy with previous rituximab experience	Obinutuzumab infusion of 1 g and second 1 g infusion 2 weeks after the first infusion, in addition to diphenhydramine, dexamethasone, acetaminophen for reactions	NA	Supportive care: ACEI, and ARBs and SGLT2 inhibitors for proteinuria, plus treatments for comorbidities like hypertension	Rituximab	9.5	6	NA

Trillini (2026)	Prospective study	Italy	Adult patients with primary membranous nephropathy and persistent nephrotic syndrome with resistance to rituximab	Three doses of obinutuzumab (100 mg) on Day 1 (100 mL saline at 25 mg/h) then 900 mg on Day 2 (250 mL saline starting 50 mg/h, titrated up to 400 mg/h; slower if prior reaction), followed by 1000 mg infusions at weeks 2 and 4 (10 mg chlorphenamine and 1 g of paracetamol and i.v. Infusion of 80 mg of methylprednisolone)	NA	NA	Calcineurin inhibitors, rituximab, and steroids	24	20	NA

Cheng (2025) (First line)	Retrospective study	China	Adult patients with primary membranous nephropathy without previous rituximab	Two IV doses of obinutuzumab 1 g given 2 weeks apart, with premedication (80 mg methylprednisolone IV) to prevent infusion reactions as initial therapy	NA	NA	NA	12	15	NA
Cheng (2025) (Second line)			Adult patients with primary membranous nephropathy with previous rituximab				Rituximab, steroids, and immunosuppressive	13	40	

*Note:* Data are presented as reported in the included studies. I and C denote intervention and comparator groups, respectively. NA indicates not available or not applicable. PLA2R, phospholipase A2 receptor; IV, intravenous; IM, intramuscular; SGLT2, sodium–glucose cotransporter‐2; MMF, mycophenolate mofetil.

Abbreviations: ACEI, angiotensin‐converting enzyme inhibitor; ARB, angiotensin receptor blocker; eGFR, estimated glomerular filtration rate.

**TABLE 2:** Baseline characteristics.

Study ID	Age, mean ± SD	Sex, male, *n* (%)	PLA2R‐poisitive	Time from disease to obinutuzumab onset, months	Concomitant diseases		eGFR (ml/min/1.73 m^2^), mean ± SD	SCr (μmol/L), mean ± SD	24 h proteinuria (g/24 h), mean ± SD	Albumin (g/L), mean ± SD	Anti‐PLA2R antibody (RU/mL), mean ± SD
					Hypertension	DM					
Xue Li (2025)	58.5 ± 2.75	20 (80)	25 (100)		15 (60)	7 (28)	97 ± 15.72	75.33 ± 22	9.95 ± 5.23	25.15 ± 6.17	157.9 ± 218.9

Su (2025)	46.25 ± 13.05	14 (70)	20 (100)	38 ± 2.8	14 (70)	2 (10)		67.54 ± 28.79	6.62 ± 5.78	26.16 ± 5.85	34.32 ± 51.07

Huixian Li (2025)	49.7 ± 13.7	11 (64.7)	17 (100)	22 ± 24.25	7 (41.2)	5 (29.4)	94.0 ± 27.8	77.5 ± 30.9	8.7 ± 3.1	22.6 ± 3.6	56.63 ± 104.9

Xu (2025)	52.77 ± 12.2	15 (75.0)	19 (95)	32.3 ± 33.47	10 (50)	3 (15)	90.1 ± 20.3	77.1 ± 23.9	6.17 ± 4.7	26.7 ± 8	120.6 ± 200.9

Luyao Li (2025)	44 ± 12.93	21 (84)		26.33 ± 22.8	13 (52)	6 (24)	95.02 ± 31.23	86.83 ± 33.4		30.08 ± 8.36	65.65 ± 93.04

Shi (2025)	G1: 57.11 ± 12.87, G2: 44.09 ± 15.15	G1: 12 (44.40), G2: 17 (73.91)	50 (100)	NA	G1: 9 (33.33), G2: 11 (47.83)	G1: 6 (22.22), G2:6 (26.09)	G1: 90.76 ± 25.81, G2:83.41 ± 29.71	G1: 66.57 ± 26.22, G2:95.27 ± 36.2	G1: 8.33 ± 3.26, G2:10.93 ± 5.46	G1: 23.53 ± 4.16, G2:24.40 ± 7.53	G1: 192.03 ± 238.2, G2:74.7 ± 66.6

Cao (2025)	48.33 ± 11.26	14 (77.8)	0 (0)	NA	NA	NA	99.2 ± 18.8	76.9 ± 19.9	7.6 ± 4.02	26.1 ± 2.7	NA

Su (2024)	50.7 ± 14.4	48 (81.4)	50 (84.7)	NA	38 (64.4)	7 (11.9)	92.2 ± 23.4	87.8 ± 33.5	4.8 ± 2.2	28.0 ± 7.2	76.33 ± 83.82

Xu (2024)	55 ± 10.7	16 (88.9)	18 (100)	63.5 ± 49.9	8 (44.4)	4 (22.2)	84.4 ± 43.9	NA	6.07 ± 3.7	27.3 ± 8	33.1 ± 37.65

Lin (2024)	51.43 ± 8.37	15 (83.3)	15 (83.3)	12.77 ± 14.72	NA	NA	NA	86.2 ± 21.8		27.2 ± 8.2	64.3 ± 132.09

Chen (2025)	58.57 ± 9.16	5 (71)	7 (100)	24 ± 21.12	NA	NA	NA	55.7 ± 27.83	11.7 ± 13.22	22.3 ± 4.4	84.16 ± 78.73

Hao (2024)	50.08 ± 12.49	6 (50)	12 (100)	NA	2 (17.7)	2 (17.7)	99.10 ± 18.46	68.75 ± 16.38	6.70 ± 3.08	25.19 ± 4.74	64.23 ± 88.65

Liu (2025)	57 ± 8.71	5 (83.3)	6 (100)	9	NA	NA	105.18 ± 32.07	NA	6.1 ± 3.33	NA	106.5 ± 125.5

Trillini (2026)	Aged between 45 and 71	18 (90)	16 (80)	13.5 ± 7.8	NA	NA	61.42 ± 13.92	NA	6.2 ± 2.75	29 ± 6.5	157.3 ± 162

Cheng (2025) (First line)	53.5 ± 16.4	6 (40)	13 (86.7)	8	5 (33.3)	3 (20)	92.7 ± 62	69.33 ± 44.2	8.2 ± 7.7	20 ± 4.9	174.2 ± 314.4
Cheng (2025) (Second line)	55.2 ± 13.3	28 (70)	26 (65)		24 (60.0)	12 (30)	72.6 ± 48.4	96.4 ± 57.4	6.3 ± 5.15	26.3 ± 6.9	21.8 ± 35.3

*Note:* Data are presented as mean ± SD or *n* (%), unless otherwise stated. NA indicates not available or not applicable. PLA2R, phospholipase A2 receptor. eGFR is reported in mL/min/1.73 m^2^, SCr in μmol/L, 24 h proteinuria in g/24 h, albumin in g/L, and anti‐PLA2R antibody level in RU/mL.

Abbreviations: DM, diabetes mellitus; eGFR, estimated glomerular filtration rate; RU, relative units; SCr, serum creatinine.

### 3.3. Risk of Bias and Quality Assessment

The quality assessment showed moderate‐to‐high methodological quality, with higher scores among comparative cohorts. On the NOS, scores ranged from 6 to 9 stars. Three cohorts achieved the maximum score (9/9) (e.g., Su 2024, Xu 2024, Cao 2025) and others scored 8/9 (Xu 2025; Xue‐Qi Li 2025), reflecting strong selection methods, well‐defined exposure/outcome ascertainment, and appropriate follow‐up; studies scoring 6/9 were predominantly single‐arm cohorts, which were not eligible for the “non‐exposed comparator” item and generally had more limited control for confounding (Table S1). Using the JBI Critical Appraisal Checklist for Case Series, quality ranged from 7/10 to 8/10, indicating generally clear reporting of inclusion criteria, case definition, participant demographics, clinical information, and outcome reporting. The most consistent limitation across case series was uncertainty about consecutive and complete inclusion, and one report had unclear statistical reporting, which reduced confidence in representativeness and interpretability (Table S2).

### 3.4. Primary Outcomes

#### 3.4.1. Obinutuzumab as First‐Line Therapy

In the first‐line obinutuzumab group, pooled analyses using a random‐effects model showed that the complete response rate across 110 patients was 0.324 with low‐to‐moderate heterogeneity (Figure 2A). When stratified by follow‐up time, the complete response rate was 0.257 at 6 months and 0.485 at 12 months, with evidence of a significant subgroup difference by time point (*p* < 0.0001). The pooled partial response rate across 110 patients was 0.508, with estimates of 0.559 at 6 months and 0.385 at 12 months, without a statistically significant subgroup difference (*p* = 0.17) (Figure 2B). The pooled CRR was high at both time points, measuring 0.813 at 6 months and 0.886 at 12 months (Figure 3A). For immunological response, pooled estimates were similarly favorable, with a response rate of 0.886 at 6 months and 0.889 at 12 months, with no evidence of a time‐based subgroup effect (*p* = 0.978) (Figure 3B). Assessment of small‐study effects using Doi plots (LFK index) suggested no asymmetry for complete response (LFK index = 1.04) (Figure S1), partial response (LFK index = 0.46) (Figure S2), and clinical remission (LFK index = 0.51) (Figure S3), while major asymmetry was observed for immunological response (LFK index = 3.26) (Figure S4).

**FIGURE 2:**
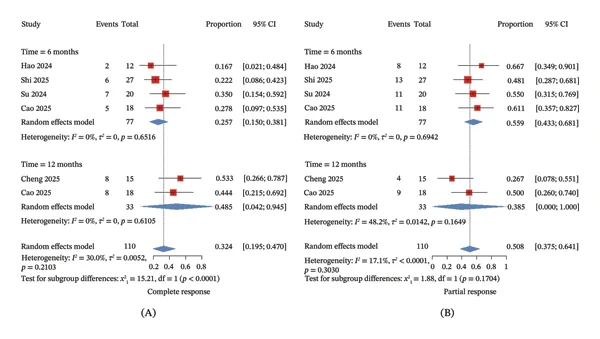
Forest plots of pooled response rates with first‐line obinutuzumab, stratified by follow‐up (6 vs. 12 months): (A) complete response; (B) partial response.

**FIGURE 3:**
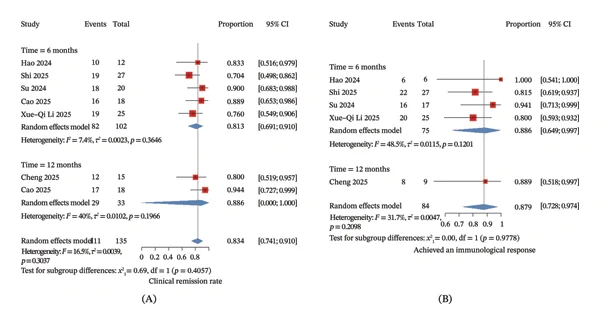
Forest plots of pooled response rates with first‐line obinutuzumab, stratified by follow‐up (6 vs. 12 months): (A) clinical remission rates; (B) immunological response.

#### 3.4.2. Obinutuzumab as Second‐Line Therapy After Prior Immunosuppressive Treatment

In the second‐line obinutuzumab group (patients previously exposed to immunosuppressive therapy), pooled random‐effects analyses demonstrated a complete response rate of 0.181 across 236 patients (Figure 4A). When stratified by follow‐up duration, complete response was 0.136 at 6 months and 0.264 at 12 months, with no significant subgroup difference between time points (*p* = 0.126). The pooled partial response rate across 236 patients was 0.547, with comparable estimates at 6 months (Proportion = 0.545) and 12 months (Proportion = 0.546), and no evidence of a subgroup effect by follow‐up duration (*p* = 0.99) (Figure 4B). The CRR was 0.638 at 6 months, increasing to 0.835 at 12 months (Figure 5A). For immunological response, pooled estimates were 0.740 at 6 months and 0.905 at 12 months, yielding an overall immunological response rate of 0.815 and with a statistically significant difference between the pooled 6‐ and 12‐month estimates (*p* = 0.013) (Figure 5B). Assessment of small‐study effects using Doi plots (LFK index) suggested no important asymmetry for complete response (LFK index = −0.8) (Figure S5) and clinical remission (LFK index = −0.86) (Figure S6), minor asymmetry for immunological response (LFK index = −1.13) (Figure S7) and for partial response (LFK index = 1.73) (Figure S8).

**FIGURE 4:**
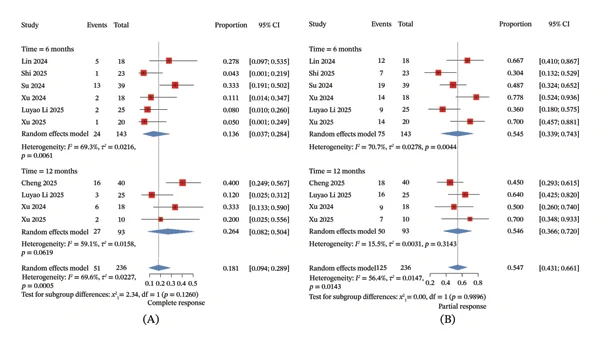
Forest plots of pooled response rates with second‐line obinutuzumab, stratified by follow‐up (6 vs. 12 months): (A) complete response; (B) partial response.

**FIGURE 5:**
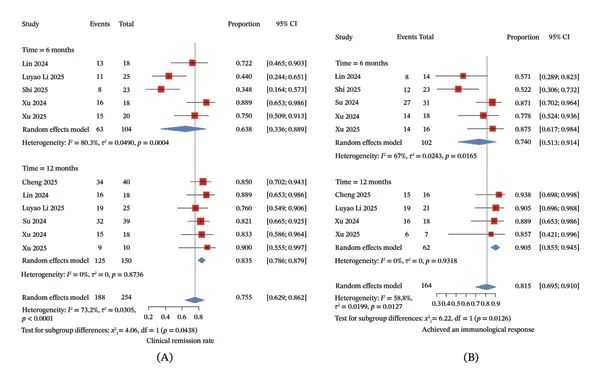
Forest plots of pooled response rates with second‐line obinutuzumab, stratified by follow‐up (6 vs. 12 months): (A) clinical remission rates; (B) immunological response.

#### 3.4.3. Rituximab‐Refractory Patients

Rituximab‐refractory meta‐analysis was restricted to retrospective Chinese studies. In the rituximab‐refractory subgroup, pooled random‐effects analyses demonstrated a modest overall complete response rate of 0.278 across 70 patients, with moderate heterogeneity (Figure 6A). When stratified by follow‐up duration, complete response was 0.361 at 6 months and 0.148 at 12 months, with no statistically significant subgroup difference by time point (*p* = 0.094). The pooled partial response rate across 70 patients was 0.543, rising from 0.471 at 6 months to 0.668 at 12 months, with nonsignificant subgroup difference (*p* = 0.07) (Figure 6B). The pooled CRR was 0.516 at 6 months and 0.845 at 12 months, with a statistically significant subgroup difference between time points (*p* = 0.004) (Figure 7A). For immunological response, the pooled proportion was high overall at 0.817 across 49 patients, with moderate heterogeneity; estimates were 0.793 at 6 months and 0.898 at 12 months, with no evidence of a time‐based subgroup effect (*p* = 0.64) (Figure 7B). Assessment of small‐study effects using Doi plots (LFK index) suggested no important asymmetry for complete response (LFK index = 0.12) (Figure S9), clinical remission (LFK index = 0.41) (Figure S10), and partial response (LFK index = −0.52) (Figure S11), whereas major asymmetry was observed for immunological response (LFK index = 2.31) (Figure S12).

**FIGURE 6:**
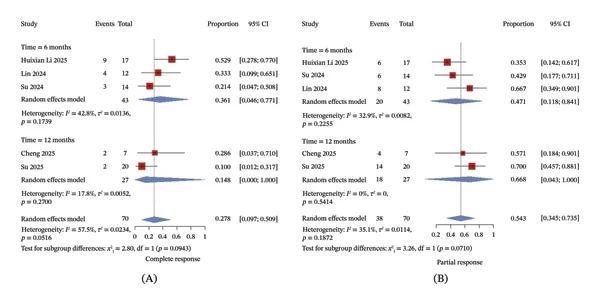
Forest plots of pooled response rates with obinutuzumab administration in rituximab‐refractory patients, stratified by follow‐up (6 vs. 12 months): (A) complete response; (B) partial response.

**FIGURE 7:**
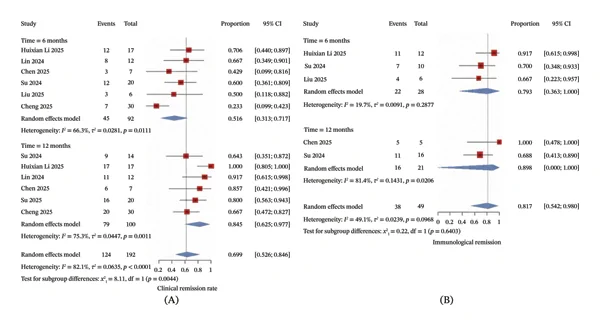
Forest plots of pooled response rates with obinutuzumab administration in rituximab‐refractory patients, stratified by follow‐up (6 vs. 12 months): (A) clinical remission rates; (B) immunological response.

The Italian prospective Trillini 2026 cohort was not included in the pooled rituximab‐refractory estimates because it differed in geography and prospective design. In this cohort, 16 of 20 patients met the combined endpoint at 12 months, including 4 CRs, and no patient relapsed after remission. In the 10 patients with 24‐month follow‐up, improvements were sustained; notably, B cells reemerged without an accompanying increase in anti‐PLA2R antibodies, proteinuria, or relapse. These findings are consistent with the direction of the retrospective Chinese data and support the hypothesis of durable immunologic disease control in some patients, though they cannot establish definitive cure.

### 3.5. Secondary Outcomes

#### 3.5.1. Obinutuzumab as First‐Line Therapy

In the first‐line obinutuzumab group, secondary outcomes demonstrated a consistent improvement in nephrotic parameters over follow‐up. Serum albumin increased significantly from baseline, with a pooled MD = 13.60 (*p* < 0.001; *I* ^2^ = 81%), and the magnitude of increase was observed as early as 3 months (MD = 10.69, *p* < 0.001), and remained higher at 6 months (MD = 14.62, *p* < 0.001) and 12 months (MD = 16.29, *p* < 0.001), without a statistically significant subgroup difference by time point (*p* = 0.16) (Figure S13). Proteinuria decreased markedly over time, with an overall pooled MD = −5.68 (*p* < 0.001; *I* ^2^ = 52%), showing reductions at 3 months (MD = −4.73, *p* < 0.001), 6 months (MD = −5.80, *p* < 0.001), and 12 months (MD = −7.28, *p* < 0.001), again with no significant subgroup difference across time points (*p* = 0.15) (Figure S14). In contrast, kidney function indices were largely unchanged: serum creatinine showed a borderline, nonsignificant increase overall (MD = 6.50, *p* = 0.05, *I* ^2^ = 0%) with similarly nonsignificant effects at 6 and 12 months (Figure S15), while eGFR demonstrated no significant change from baseline (MD = −0.62, *p* = 0.91; *I* ^2^ = 60%), with nonsignificant estimates at both 6 months (MD = 0.68, *p* = 0.93) and 12 months (MD = −5.30, *p* = 0.45), and no evidence of a subgroup effect by follow‐up duration (*p* = 0.56) (Figure S16).

#### 3.5.2. Obinutuzumab as Second‐Line Therapy After Prior Immunosuppressive Treatment

In the second‐line obinutuzumab group, secondary outcomes showed significant improvement in nephrotic parameters, particularly serum albumin and proteinuria, while no statistically significant overall change in serum creatinine or eGFR was detected. Serum albumin increased significantly from baseline across 224 patients, with an overall pooled MD = 11.64 (*p* < 0.001; *I* ^2^ = 70%). This increase was evident at 3 months (MD = 7.12, *p* = 0.0005), became more pronounced at 6 months (MD = 11.43, *p* < 0.001), and was sustained at 12 months (MD = 13.85, *p* < 0.001), without a significant subgroup effect by follow‐up duration (*p* = 0.13) (Figure S17). Proteinuria decreased significantly over time, with an overall pooled MD = −3.90 (*p* < 0.001; *I* ^2^ = 32%), showing reductions at 3 months (MD = −2.70, *p* = 0.02), 6 months (MD = −3.13, *p* < 0.001), and 12 months (MD = −5.13, *p* < 0.001), with evidence of a time‐related subgroup effect (*p* = 0.04) consistent with greater reductions at longer follow‐up (Figure S18). In contrast, serum creatinine showed no significant overall change from baseline (MD = −5.47, *p* = 0.1; *I* ^2^ = 0%), with nonsignificant estimates at both 6 months (MD = −4.09, *p* = 0.32) and 12 months (MD = −8.13, *p* = 0.16) and no subgroup difference by time point (*p* = 0.57) (Figure S19). Similarly, eGFR did not change significantly overall (MD = 7.02, *p* = 0.08; *I* ^2^ = 0%), with borderline improvement at 6 months (MD = 9.80, *p* = 0.05) and no significant change at 12 months (MD = 2.18, *p* = 0.74), without evidence of a subgroup effect (*p* = 0.36) (Figure S20).

#### 3.5.3. Rituximab‐Refractory Patients

In the rituximab‐refractory subgroup, secondary outcomes suggested improvement in nephrotic parameters driven primarily by longer follow‐up, while renal function remained largely unchanged. Serum albumin showed an overall nonsignificant increase from baseline (MD = 9.35, *p* = 0.08), though this was accompanied by marked heterogeneity (*I* ^2^ = 96%) (Figure S21). Notably, there was a significant subgroup difference by time point (*p* < 0.0001): at 6 months, albumin change was minimal and nonsignificant (MD = −0.31, *p* = 0.82), whereas at 12 months, a significant increase was observed (MD = 13.92, *p* < 0.001, *I* ^2^ = 0%). Proteinuria decreased overall (MD = −4.33, *p* = 0.02) with substantial heterogeneity (*I* ^2^ = 71%) and a statistically significant overall effect (*p* = 0.02) (Figure S22). This reduction was not evident at 6 months (MD = −1.32, *p* = 0.31), but became significant at 12 months (MD = −6.3, *p* < 0.007), with a nearly significant subgroup effect by time point (*p* = 0.06). In contrast, serum creatinine did not change significantly from baseline (MD = −6.73, *p* = 0.28, *I* ^2^ = 0%), with nonsignificant estimates at both 6 months (MD = −14.00, *p* = 0.16) and 12 months (MD = −2.11, *p* = 0.79), and no evidence of a time‐based subgroup difference (*p* = 0.35) (Figure S23).

## 4. Discussion

In this systematic review and meta‐analysis of 15 studies, we synthesized the available clinical evidence on obinutuzumab for PMN and quantified outcomes by treatment line. Across subgroups, obinutuzumab was associated with high rates of immunological response and progressively increasing clinical remission with longer follow‐up. This course is coherent with the contemporary antigen‐specific model of PMN, in which circulating autoantibodies, most commonly to PLA2R, drive subepithelial immune deposits and podocyte injury [3, 7, 39]. Importantly, the treatment‐line strata represent clinically distinct patient populations rather than randomized comparisons. Consequently, differences in pooled response proportions between strata should not be interpreted as evidence that treatment line itself modifies obinutuzumab efficacy.

Treatment‐line stratification revealed clinically relevant gradients. In treatment‐naïve patients, the pooled complete remission rate increased from about one‐quarter at 6 months to nearly one‐half at 12 months, while pooled clinical remission either complete or PRs was already high at 6 months with sustained favorable outcomes after 12 months. Secondary outcomes were consistent with these estimates: serum albumin increased and proteinuria decreased substantially, whereas kidney function measures were largely stable. Importantly, immunological response rates were high by 6 months and remained high at 12 months, underscoring the well‐described lag between antibody depletion and full clinical remission of proteinuria [7, 8, 10].

Importantly, the higher clinical remission proportions observed at later follow‐up cannot be attributed solely to increasing pharmacological efficacy of obinutuzumab. In PMN, immunological activity commonly improves before proteinuria resolves because restoration of the glomerular filtration barrier may require additional time after suppression of pathogenic autoantibodies. Furthermore, spontaneous remission is a recognized component of the natural history of PMN, particularly in patients with declining anti‐PLA2R antibody levels. This is especially relevant to the treatment‐naïve cohorts in our synthesis. Because these studies were predominantly uncontrolled, the respective contributions of obinutuzumab, optimized supportive therapy, spontaneous remission, and delayed clinical expression of an earlier immunological response cannot be separated [40, 41].

In patients treated with obinutuzumab as second‐line therapy, complete remission was less frequent early and between‐study heterogeneity was substantial. This is unsurprising given variability in prior regimens (CNIs, alkylating agents, steroids, and/or anti‐CD20 therapy), baseline immunologic activity, and chronicity on biopsy. Moreover, pooled clinical remission increased significantly by 12 months, and immunological response was robust with a statistically significant improvement in antibody negativity at 12 months compared with 6 months. Clinically, these data support obinutuzumab as a potential rescue option in previously treated PMN, while reinforcing that response assessment should extend beyond the early 6‐month time point whenever clinically safe [10].

The rituximab‐refractory subgroup is particularly practice relevant. The pooled clinical remission was modest at 6 months but rose substantially by 12 months, while immunological response remained high. Although confidence intervals were wide due to limited sample sizes, this signal aligns with multiple reports describing remission and antibody depletion after obinutuzumab in rituximab‐exposed or rituximab‐nonresponsive PMN, including early Kidney International Reports experience and more recent cohorts and case series [19, 31, 32, 42].

A biologically plausible explanation for these observations lies in the functional differences between obinutuzumab and type I anti‐CD20 antibodies. Obinutuzumab is a glycoengineered type II anti‐CD20 monoclonal antibody designed to enhance antibody‐dependent cellular cytotoxicity and phagocytosis and to induce direct B‐cell death; preclinical comparisons demonstrate distinct effector mechanisms and, in multiple models, stronger cytotoxic activity than rituximab and ofatumumab [16, 17].

Recent evidence syntheses are broadly concordant with our findings, though methodological differences limit direct numerical comparison. Lurie et al. support the potential role of obinutuzumab in PMN with higher odds of total clinical remission at 6 and 12 months, higher odds of complete clinical remission at 12 months, and a higher rate of immunologic remission in favor of obinutuzumab, while emphasizing the small and geographically restricted evidence base. [43]. Atay et al., using pooled case‐level data from 89 patients in 19 publications, reported overall clinical and immunological remission rates of 83% and 88.7%, respectively [44]. A more recent single‐arm meta‐analysis by Cong et al. of 12 studies and 222 patients with refractory MN reported clinical remission, CR, PR, and immunological remission rates of 86%, 31%, 55%, and 88%, respectively [45]. These estimates are particularly close to our refractory‐group complete and partial response estimates of 27.8% and 54.3% and to our 12‐month clinical and immunological remission estimates of 84.5% and 89.8%. Differences in inclusion criteria, treatment history, follow‐up duration, geographic composition, and analytical approach likely explain the remaining variation between reviews. These findings are broadly consistent with our treatment‐line‐specific synthesis, in which obinutuzumab showed high immunologic response and progressively increasing clinical remission over time, especially in previously treated and rituximab‐refractory patients. These observations suggest that obinutuzumab may represent a therapeutic option for patients with persistent immune activity or an inadequate response to rituximab, though comparative effectiveness cannot be established from these data.

### 4.1. Clinical Implications

Positioning these findings within current PMN management highlights potential clinical implications. KDIGO recommends risk‐stratified immunosuppression using rituximab, cyclophosphamide plus glucocorticoids, or CNI‐based regimens, with treatment selection guided by patient risk and comorbidity profile [10]. Clinical trials illustrate the efficacy of established approaches and the importance of context: rituximab achieved durable remission compared with cyclosporine in MENTOR, cyclic cyclophosphamide‐steroid therapy outperformed tacrolimus‐rituximab in STARMEN, and RI‐CYCLO did not show a clear signal of superiority for rituximab over the cyclic regimen in its pilot design [13–15]. Against this backdrop, our pooled remission estimates suggest that obinutuzumab has potential as an alternative B‐cell‐targeted strategy, particularly in relapsed or refractory cases, but direct comparative evidence is still limited.

From a practical standpoint, the consistently notable immunological response across treatment lines reinforces the value of serology‐guided monitoring. Anti‐PLA2R antibody kinetics provide prognostic information and can help distinguish persistent immune activity from delayed clinical resolution of proteinuria, informing whether additional immunosuppression is warranted or observation is reasonable [7, 8, 10]. Future studies should harmonize reporting of baseline antibody titers, assay platforms and thresholds, and the definition and timing of immunological response, and should report time‐to‐immunologic remission alongside time‐to‐clinical remission. Such standardization would reduce heterogeneity and enable more reliable, response‐adapted retreatment strategies.

The economic implications of broader obinutuzumab use remain uncertain and are likely to be highly setting dependent. Acquisition costs vary according to dose and number of infusions, local pricing, reimbursement, and the availability of rituximab biosimilars [31, 46]. Published PMN reports illustrate this variability, with some local treatment schedules suggesting comparable or lower acquisition costs for obinutuzumab [31], whereas analyses based on the four‐dose MAJESTY regimen suggest substantially higher drug costs than conventional rituximab schedules, particularly when biosimilars are available [46]. Drug acquisition cost alone, however, does not establish cost‐effectiveness. A comprehensive economic analysis would also need to incorporate infusion costs, retreatment, monitoring, adverse events, rescue therapy, relapse, hospitalization, potential avoidance of chronic immunosuppression, and long‐term kidney outcomes. Dedicated PMN‐specific cost‐utility analyses are therefore required before broad first‐line adoption can be considered economically justified [46].

Safety could not be reliably characterized in the present synthesis because adverse‐event reporting was inconsistent across studies, sample sizes were small, and follow‐up was generally limited. Therefore, our findings should not be interpreted as establishing an acceptable or comparative safety profile for obinutuzumab in PMN. Class‐related concerns include infusion reactions, infection, neutropenia, and hypogammaglobulinemia. Recent evidence provides additional context: a single‐arm meta‐analysis of refractory MN reported a pooled adverse‐event rate of 29% and included two fatal pneumonia‐related events [45], while the MAJESTY randomized trial reported grade ≥ 3 adverse events in 22% and serious adverse events in 17% of obinutuzumab‐treated patients [47]. Nonetheless, randomized lupus nephritis trial and accumulating kidney‐specific observational experience suggest that obinutuzumab can be administered with an acceptable safety profile when patients are appropriately selected and monitored, while acknowledging class risks of anti‐CD20 therapy, including infusion reactions, infections, and hypogammaglobulinemia [18, 19, 31, 42, 48]. In practice, careful vaccination planning, infection risk mitigation, and monitoring of immunoglobulin levels, especially with repeated dosing, are essential to avoid unexpected reactions.

### 4.2. Limitations and Recommendations

This study has several limitations to consider. First, the evidence base is dominated by retrospective cohorts and case series; therefore, the pooled estimates cannot establish causality and remain susceptible to selection bias, residual confounding, regression to the mean, time effects, placebo, concomitant therapies, and spontaneous or delayed remission. Second, obinutuzumab regimens (dose, number of infusions, and retreatment policies) and concomitant therapies varied, contributing to heterogeneity, particularly in previously treated populations. Third, follow‐up was often limited to 12 months; our subgroup analyses indicate that remission rates can continue to rise between 6 and 12 months. Fourth, most pooled data came from China, and the only Italian prospective cohort was therefore considered separately in the rituximab‐refractory analysis. Outcome definitions, anti‐PLA2R assay thresholds, obinutuzumab regimens, follow‐up schedules, and adverse‐event reporting varied across studies. Immunological remission also requires particularly cautious interpretation as included studies used different anti‐PLA2R assay platforms and study‐specific thresholds for seronegativity. Consequently, an event classified as immunological remission did not necessarily represent an identical serological state across studies. This concern is reinforced by evidence of small‐study asymmetry in some immunological response analyses. Fifth, the apparent stability of kidney function measures should not be interpreted as evidence of renoprotection. Most included patients had relatively preserved eGFR at baseline, sample sizes were small, and follow‐up was generally too short to robustly evaluate hard kidney outcomes or clinically meaningful eGFR decline. Finally, evidence of small‐study effects was suggested for some immunological response analyses, emphasizing the need for larger, prospectively designed studies with standardized outcomes.

However, this treatment‐line‐specific synthesis indicates that obinutuzumab is associated with substantial immunologic activity and clinical remission that strengthens with longer follow‐up, including in rituximab‐refractory disease. Prospective controlled trials are now needed to define comparative effectiveness, optimal dosing and retreatment strategies, durability of response, and long‐term kidney outcomes. Randomized evaluation in PMN is ongoing and should help clarify where obinutuzumab best fits alongside established regimens [49].

## 5. Conclusion

In conclusion, obinutuzumab‐treated patients with PMN demonstrated high observed clinical and immunological response proportions across different treatment contexts. Higher clinical remission proportions at later follow‐up should be interpreted in the context of the recognized delay between immunological response and proteinuria resolution, longer observation time, and the possibility of spontaneous remission, rather than as evidence that treatment efficacy increases over time. Because the treatment‐line strata comprised clinically heterogeneous, nonrandomized populations, differences between strata cannot establish differential efficacy or treatment‐line effect modification. Similarly, the absence of substantial short‐term changes in kidney function measures should not be interpreted as evidence of renoprotection, and the available data are insufficient to establish the safety profile of obinutuzumab in PMN. Further comparative evidence, particularly against rituximab, together with longer term safety, kidney outcome, and cost‐effectiveness data, is needed to define its optimal position in PMN treatment.

## Author Contributions

Abdelrahman M. Elettreby: conceptualization, methodology, literature search, study selection, data extraction, formal analysis, visualization, writing–original draft, and writing–review and editing. Ahmed A. Abo Elnaga: conceptualization, methodology, literature search, study selection, data extraction, validation, and writing–review and editing. Faisal A. Al‐Harbi: methodology, validation, interpretation of results, and writing–review and editing. Sayed Sayem: methodology, statistical analyses, validation, software and writing. Tamara Aburiash: literature search, study selection, data extraction, and writing–review and editing. Alam Eldin Musa Mustafa: validation, interpretation of results, supervision, and writing–review and editing. Dina Khodeer: validation, interpretation of results, supervision, and writing–review and editing. Ahmed Mohamed Aboelkheir: methodology, data verification, interpretation of results, and writing–review and editing. Mostafa Gamal: conceptualization, methodology, supervision, project administration, interpretation of results, and writing–review and editing.

## Funding

The authors of this study did not receive any grants or funding for conducting this study from any organization or institution.

## Disclosure

All authors critically reviewed and approved the final version of the manuscript and agreed to be accountable for all aspects of the work.

## Ethics Statement

No ethical approval was required for this study, as it is a PRISMA‐compliant systematic review and meta‐analysis. The protocol of this study was prospectively registered with PROSPERO to maintain transparency.

## Conflicts of Interest

The authors declare no conflicts of interest.

## Supporting Information

Additional supporting information can be found online in the Supporting Information section.

## Supporting information


**Supporting Information 1** Figure S1. Doi plot assessing small‐study effects for complete response in the first‐line obinutuzumab group. Figure S2. Doi plot assessing small‐study effects for partial response in the first‐line obinutuzumab group. Figure S3. Doi plot assessing small‐study effects for clinical remission in the first‐line obinutuzumab group. Figure S4. Doi plot assessing small‐study effects for immunological response in the first‐line obinutuzumab group. Figure S5. Doi plot assessing small‐study effects for complete response in the second‐line obinutuzumab group. Figure S6. Doi plot assessing small‐study effects for clinical remission in the second‐line obinutuzumab group. Figure S7. Doi plot assessing small‐study effects for immunological response in the second‐line obinutuzumab group. Figure S8. Doi plot assessing small‐study effects for partial response in the second‐line obinutuzumab group. Figure S9. Doi plot assessing small‐study effects for complete response in the rituximab‐refractory group. Figure S10. Doi plot assessing small‐study effects for clinical remission in the rituximab‐refractory group. Figure S11. Doi plot assessing small‐study effects for partial response in the rituximab‐refractory group. Figure S12. Doi plot assessing small‐study effects for immunological response in the rituximab‐refractory group. Figure S13. Forest plot of change in serum albumin over time in the first‐line obinutuzumab group. Figure S14. Forest plot of change in proteinuria over time in the first‐line obinutuzumab group. Figure S15. Forest plot of change in serum creatinine over time in the first‐line obinutuzumab group. Figure S16. Forest plot of change in eGFR over time in the first‐line obinutuzumab group. Figure S17. Forest plot of change in serum albumin over time in the second‐line obinutuzumab group. Figure S18. Forest plot of change in proteinuria over time in the second‐line obinutuzumab group. Figure S19. Forest plot of change in serum creatinine over time in the second‐line obinutuzumab group. Figure S20. Forest plot of change in eGFR over time in the second‐line obinutuzumab group. Figure S21. Forest plot of change in serum albumin over time in the rituximab‐refractory group. Figure S22. Forest plot of change in proteinuria over time in the rituximab‐refractory group. Figure S23. Forest plot of change in serum creatinine over time in the rituximab‐refractory group.

**Supporting Information 2** Table S1. Newcastle–Ottawa Scale (NOS) quality assessment of included cohort studies. Table S2. JBI Critical Appraisal Checklist assessment for included case series.

**Supporting Information 3** PRISMA_filled.

## Data Availability

The data that support the findings of this study are available from the corresponding author upon reasonable request.
