# Supplementary material for: Treatment‐Line‐Specific Outcomes of Obinutuzumab in Membranous Nephropathy: A Systematic Review and Meta‐Analysis
Source: Int J Nephrol. 2026 Nov 9;2026:6372570. doi: 10.1155/ijne/6372570 (PMC13746314; doi:10.1155/ijne/6372570)
Supplement: Supplementary file 1 [file IJNE-2026-6372570-s001.docx]

Figure S1

Figure S2

Figure S3

Figure S4

Figure S5

Figure S6

Figure S7

Figure S8

Figure S9

Figure S10

Figure S11

Figure S12

Figure S13

Figure S14

Figure S15

Figure S16

Figure S17

Figure S18

Figure S19

Figure S20

Figure S21

Figure S22

Figure S23
