# Supplementary material for: Treatment‐Line‐Specific Outcomes of Obinutuzumab in Membranous Nephropathy: A Systematic Review and Meta‐Analysis
Source: Int J Nephrol. 2026 Nov 9;2026:6372570. doi: 10.1155/ijne/6372570 (PMC13746314; doi:10.1155/ijne/6372570)
Supplement: Supplementary file 2 [file IJNE-2026-6372570-s002.docx]

**Table S1.** NOS (Cohort) assessment for cohort studies

| **Study** | **Rep.** | **Non-exp.** | **Ascertain exp.** | **Outcome absent** | **Comp (0-2)** | **Outcome assess** | **FU long** | **FU adequate** | **Total/9** |
| --- | --- | --- | --- | --- | --- | --- | --- | --- | --- |
| Chen 2025 | 1 | N/A | 1 | 1 | 0 | 1 | 1 | 1 | 6 |
| Cheng 2025 | 1 | 1 | 1 | 1 | 0 | 1 | 1 | 1 | 7 |
| Su 2024 | 1 | 1 | 1 | 1 | 2 | 1 | 1 | 1 | 9 |
| Su 2025 | 1 | N/A | 1 | 1 | 0 | 1 | 1 | 1 | 6 |
| Luyao Li 2025 | 1 | N/A | 1 | 1 | 0 | 1 | 1 | 1 | 6 |
| Lin 2024 | 1 | N/A | 1 | 1 | 0 | 1 | 1 | 1 | 6 |
| Trillini 2026 | 1 | N/A | 1 | 1 | 0 | 1 | 1 | 1 | 6 |
| Xu 2024 | 1 | 1 | 1 | 1 | 2 | 1 | 1 | 1 | 9 |
| Cao 2025 | 1 | 1 | 1 | 1 | 2 | 1 | 1 | 1 | 9 |
| Xu 2025 | 1 | 1 | 1 | 1 | 1 | 1 | 1 | 1 | 8 |
| Xue-Qi Li 2025 | 1 | 1 | 1 | 1 | 1 | 1 | 1 | 1 | 8 |
| Shi 2025 | 1 | 1 | 1 | 1 | 0 | 1 | 1 | 1 | 7 |

NOS items were scored as 1 (star), 0 (no star), or N/A (single-arm cohorts without a non-exposed comparator).

**Table S2.** JBI checklist assessment for case series

| **Study** | **Inclusion criteria** | **Condition measured** | **Case ID valid** | **Consecutive** | **Complete** | **Demographics** | **Clinical info** | **Outcomes/follow-up** | **Setting described** | **Stats appropriate** | **Yes (n/10)** |
| --- | --- | --- | --- | --- | --- | --- | --- | --- | --- | --- | --- |
| Hao 2024 | Yes | Yes | Yes | Unclear | Unclear | Yes | Yes | Yes | Yes | Yes | 8/10 |
| Huixian Li 2025 | Yes | Yes | Yes | Unclear | Unclear | Yes | Yes | Yes | Yes | Yes | 8/10 |
| Liu 2025 | Yes | Yes | Yes | Unclear | Unclear | Yes | Yes | Yes | Yes | Unclear | 7/10 |

When reporting was unclear (e.g., losses to follow-up), the conservative option (no star / Unclear) was used.
